# Nano-Hydroxyapatite Gel and Its Effects on Remineralization of Artificial Carious Lesions

**DOI:** 10.1155/2021/7256056

**Published:** 2021-11-08

**Authors:** Apa Juntavee, Niwut Juntavee, Ainaj Nuñez Sinagpulo

**Affiliations:** ^1^Division of Pediatric Dentistry, Department of Preventive Dentistry, Faculty of Dentistry, Khon Kaen University, Khon Kaen 40002, Thailand; ^2^Department of Prosthodontics, Faculty of Dentistry, Khon Kaen University, Khon Kaen 40002, Thailand; ^3^Division of Pediatric Dentistry and Biomaterials Research, Faculty of Dentistry, Khon Kaen University, Khon Kaen 40002, Thailand

## Abstract

**Introduction:**

Nano-hydroxyapatite gel (NHG) has never been investigated for enamel remineralization. This study evaluated the effects of two concentrations of NHG on remineralization of an artificial carious lesion in comparison with nano-HA toothpaste (NHT) and fluoride varnish (FV).

**Materials and Methods:**

Carious lesions were prepared on 100 enamel samples and divided into 5 groups: FV, NHT, 20% NHG, and 30% NHG. One untreated (NT) group was left as control. The hardness of the surface was evaluated before, during, and after remineralization. Microhardness at various phases and the percent recovery of hardness (%HR) were determined and analyzed with ANOVA. Polarized-light micrographs (PLM) were evaluated for depth of the carious lesion.

**Results:**

Significantly different remineralization capability was indicated for tested agents (*p* < 0.05). NHT was significantly capable of remineralization greater than NHG, FV, and NT (*p* < 0.05). No noticeable difference in %HR between 20% NHG and 30% NHG (*p* > 0.05) was found. Decreasing in the depth of caries lesion was notified by PLM as applying either NHT or NHG as greater than FV, with no reduction in the depth for NT.

**Conclusions:**

Nano-HA both in toothpaste and gel form was capable of remineralization better than fluoride varnish. Comparable remineralization of 20% versus 30% NHG was evidenced. NHG for both concentrations was recommended as a capable remineralizing agent for caries remineralization. *Clinical Significance:* This study indicated that an application of nano-HA gel is an attractive route to deliver the material and can be more effective and less toxic than conventional formulations and provide its effectiveness directly at the site of action, especially for a noncooperative young child and medicinally intimidated patients who may face with inconvenience in using toothbrush and toothpaste for hygiene control.

## 1. Introduction

The contemporary approach in a carious investigation has lately been relocated to the advancement of methods for caries detection at the early phase of the disease to provide a noninvasive therapeutic procedure [1]. Enamel demineralization concerns the process of losing inorganic ions from the crystalline hydroxyapatite (HA) complex. The process for the inorganic ions to repair the crystalline HA is defined as remineralization [1]. The demineralization occurs during the acidic challenge and remineralization processes replace concurrently under neutralization balanced with calcium and phosphate available on the tooth surface [2]. However, a noticeable amount of inorganic ions was absent from the crystalline HA complex beyond any devastation in its lattice. Impairment of the HA crystal lattice, finally, provokes cavities. Demineralization is considered a repairable process, mostly in shallow enamel lesions. Usually, the partially demineralized crystalline HA network of the tooth possibly regenerates to its primordial stage if it is exposed to the oral conditions that promote remineralization [3]. Given an appropriate alteration of oral situations, remineralization shall become dominant, triggering the reparation of the lesion. To establish a carious remineralized process, a significant increase in the ion concentration of calcium or fluoride in the saliva needs to be legitimate [4]. The treatment by remineralization was suggested to be a potential development in the clinical approach for the management of dental caries nowadays [5, 6]. Remineralizing agents have been explored to restore demineralized enamel, and biomimetic strategies have been proposed for the treatment of enamel demineralization. Historically, fluoride has been the first attempt to obtain enamel remineralization [7–9]. Subsequently, new compounds have been recently introduced, for instance, casein phospho-peptide and amorphous calcium-phosphate [10] and biomimicry hydroxyapatite [11], all showing promising results. Although fluoride is further stable and is more acid-resistant than HA crystal, there is a restricted remineralized process due to the quantity of calcium and phosphate ions obtainable in the oral fluid [5, 12]. Moreover, the capability of fluoride just endeavors to reduce the disintegration of apatite instead of encouraging remineralization of the inorganic loss from the crystalline HA [13–16].

Hydroxyapatite, the main mineral composition found in the bone and tooth of human beings, is the utmost stable calcium-phosphate compound under physiologic conditions. The application of synthetic HA for biomedical materials was reported [17]. It is observed that the fundamental structure of the crystalline enamel consisted of HA particles, approximately 20–40 nm in size [18]. Nevertheless, once the enamel fully matures, the protein components virtually degenerate. Therefore, the enamel cannot be biologically remodeled [13, 19]. The synthetic HA was suggested to be utilized for the process of remineralization [19, 20]. Nano-HA was found to possess similar properties as a biological apatite [18]. However, it exhibited much higher bioactivity and enhanced mechanical properties but indicated higher resorption compared to larger HA [21]. Upon acidic circumstances, nano-HA can considerably be enhancing the remineralization potential by increasing the dissemination of mineral ions to the center of the lesion [22, 23]. It additionally adheres to the demineralized porosities and establishes a constant apatite film [24, 25]. Moreover, nano-HA showed an extreme affinity to enamel and was considered to be one of the potent biologically compatible and bioactive materials that accomplished wide-ranging interests in dental medicine over the years [26, 27].

The toothpaste containing nano-HA is capable of assisting remineralization as well as toothpaste containing fluoride, thus inhibiting the demineralization process. An in vitro pH-cycling study on the effect of nano-HA toothpaste on remineralization revealed that the use of nano-HA can reduce the caries advancement and increase the hardness of enamel [28]. The adding of nano-HA into the mouth rinse containing sodium fluoride results in a synergic reaction on the caries remineralization of the enamel [29]. The topical application of nano-HA also indicates an attractive route to deliver the material in the oral cavity. It can be more effective than conventional formulations and provide its effectiveness directly at the site of action [30]. The application of surfactants as sodium carboxymethyl cellulose (SCMC) is a widely adopted method [31] and prepared from agricultural products that address environmental and economic concerns [32]. There is a rare study related to nano-HA gel and enamel remineralization. This study is directed at comparing the potential in remineralization of nano-HA gel on artificial caries lesions with toothpaste containing nano-HA and fluoride varnish. The null hypothesis was no significantly different capability in the remineralization among nano-HA gel, nano-HA toothpaste, and fluoride varnish on artificial caries.

## 2. Materials and Methods

The experiment was pursued in vitro with the ethical approval on the protection of human subjects and animals in research from the Ethics Research Committee for Human (Approval Number: HE 592416) to protect animal and human subjects, by utilizing the CRIS format as the experimental guideline.

### 2.1. Enamel Specimen Preparation

**Figure 1:**
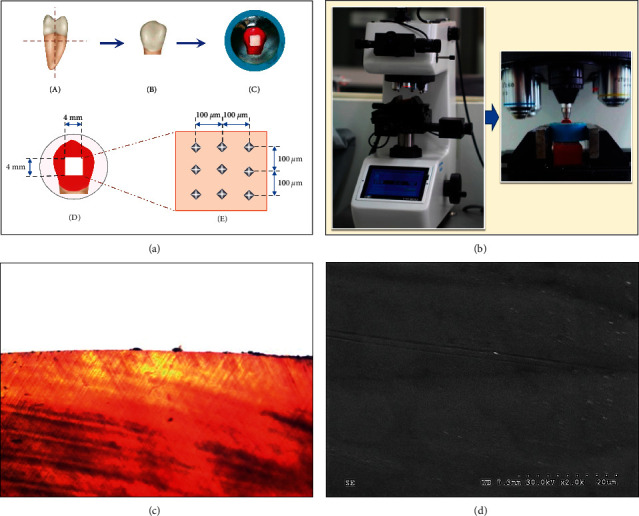
(a) The second premolar teeth (A) were cut in the horizontal direction below cementum-enamel junction for 1 mm and vertically sectioned at the central groove in the mesiodistal direction (A). The crown (B) was embedded in the resin (C) and flattened the surface for 4 × 4 mm^2^ (D). The hardness was evaluated on the flatten area at 100 *μ*m far from each indentation (E) in the Vickers hardness testing machine (b). Polarized light micrograph (c) and scanning electron micrograph (X2.0K) (d) were used to verify the intact enamel for the tested group.

One hundred extracted human bicuspids free from carious lesions, white spots, craze lines, or abnormal developments were included in this experiment. The sample size was computed based on the previous study [33] using the Piface program version 1.76 [34] with a power of test 90%, a significance level of 0.05, and a two-tailed analysis. Before any extraction, informed consent was written by the patients and their parents. They were kept in 0.1% thymol solution and washed out with deionized water before being used in the experiment. The roots were isolated from the crowns (Figure 1(a)) and sectioned into two halves by a diamond cutting blade under continuous water cooling in a precise sectioning apparatus (Mecatome-T180, Presi, Eybens, France; Figure 1(a)). The specimens were invested in the epoxy resin blank exposing enamel above the epoxy resin materials. The enamel surfaces of the specimens were coated with the acidic resistance varnish (Revlon®, New York, NY, USA), leaving the opening window of 4 × 4 mm (Figure 1(a)). The uncoated enamel surface was flattened using a silicon carbide abrasive paper (Buehler, Tokyo, Japan) with grit #1000 ⟶ #2000 ⟶ #4000 sequentially, in a finishing apparatus (Ecomet, Buehler, Tokyo, Japan; Figure 1(a)). The specimens were washed and immersed in 37°C deionized water for 1 day [35].

### 2.2. Preparation Artificial Caries Lesion

The synthetic polymer gel for preparing simulated caries lesion was produced for demineralized gel, comprising Carbopol-907 (BF Goodrich, Cleveland, OH, USA) of 20 g/L, hydroxyapatite of 500 mg/L, and lactic acid of 0.1%, and equilibrated the pH to 5.0 using sodium hydroxide [36]. All samples were immersed in a demineralization solution in a humid chamber for 12 hours to produce a simulated carious area on enamel and washed with deionized water to eradicate the demineralization solution from the specimen.

### 2.3. Remineralization Procedure

The samples were statistically determined [33, 34] and aimlessly divided into five groups (*n* = 20), to be manipulated upon various remineralizing materials (Table 1), as follows:

  Group FV: the samples were applied with fluoride varnish (FV, Duraphat®, Colgate Palmolive, Guildford, UK) using a microbrush and left on the enamel surface for 12 hours and then removed using cotton tips immersed in deionized water without rubbing.
  Group NHT: The samples were applied with the toothpaste containing nano-HA (NHT, Apagard®, Sangi, Tokyo, Japan) in the form of a slurry that was prepared following EN ISO 11609 by diluting the toothpaste containing nano-HA in three parts (1:3) to deionized water. The samples were applied by brushing twice daily using an electric toothbrush for 5 seconds in conjunction with an additional exposure time with the solution for 115 seconds, rendering a total exposure time for 120 seconds. The samples were cleaned with deionized water for 10 seconds after brushing.
  Group 20% NHG: The samples were applied with the 20% concentration of nano-HA gel (20% NHG, Biomaterials Research, KKU) that was prepared by dissolving the nano-HA powder (Sigma-Aldrich, St. Louis, MO, USA) with a computed volume of deionized water and weight of sodium carboxymethylcellulose (SCMC) to achieve the 20% in concentration. The samples were applied with 20% NHG and left on the enamel surface for 4 minutes twice daily and then rinsed thoroughly with deionized water.
  Group 30% NHT: The samples were applied with the 30% concentration of nano-HA gel (30% NHG, Biomaterials Research, KKU) that was prepared in the same technique as described for 20% NHG but reaching 30% in concentration and then applied on the enamel surface for 4 minutes twice daily before rinsing thoroughly with deionized water.
  Group NT: The samples were left without any application in the deionized water (NT) to serve as a control group.

After the application of the agents, all groups were immersed in deionized water in different containers for 30 cycles and placed in an incubator with a temperature of 37°C. The deionized water was replaced on every new cycle [35].

### 2.4. Evaluation of the Surface Microhardness

The surface hardness was blindly determined by a calibrated evaluator before applying with the demineralized agent (*H*_B_), after applying with the demineralized agent (*H*_D_), and after applying with remineralized materials (*H*_A_). An indent was aimlessly determined at 100 *μ*m far from the others (Figure 1(a)) by indentation with Vickers microhardness indenter at 100 g loading with dwelling time for 15 seconds using a fully automatic digital automatic hardness tester (FM-800, Future-tech, Tokyo, Japan) to determine the Vickers hardness number (VHN; Figure 1(b)) and then computed for the percent of hardness recovery (%HR) as follows [35]:

(1) $$\begin{array}{c} \%\text{HR}=\frac{H_{\text{A}}-H_{\text{D}}}{H_{\text{B}}-H_{\text{D}}}\times 100. \end{array}$$

### 2.5. Microscopic Evaluation

#### 2.5.1. Polarized Light Microscopy

The sample in each group was sectioned in a longitudinal direction for the thickness of 250 *μ*m, washed with deionized water, and then examined with a polarized light microscope (PLM, at 10X magnification, Eclipse-80i, Nikon, Kanagawa, Japan). The PLM (at 10X magnification) of the flawless enamel surface was investigated for use as a reference for comparison with other groups (Figure 1(c)).

#### 2.5.2. Scanning Electron Microscopy

The sample in each group was covered with gold with a sputtered apparatus (Emitech-K500X, Quorum Technologies, Asford, UK), further evaluated for surface variation with the scanning electron microscope (SEM, S-3000N, Hitachi, Tokyo, Japan), and compared to the SEM micrograph of the normal flawless enamel surface at the magnification of 2.0 K (Figure 1(d)).

#### 2.5.3. Evaluation of Crystal Structure

The specimens from each group were aimlessly adopted and crushed into very fine particles and then determined for the crystal structure by using the X-ray diffraction machine (PANalytical B.V., Almelo, Netherland). The finely ground particles were inspected using Cu k-alpha (Cu K *α*) spectrums by 40 kV, 30 mA from 10 to 60° of a diffraction angle (2*θ* degree). The crystalline microstructures were analyzed by matching with the standard references of diffraction and excused for the peak intensity using X'pert-plus software (PANalytical B.V., Almelo, Netherland) with stepwise of 0.02° for every 2 seconds. The size of the crystal was evaluated by the formula derived by Scherrer [28], as shown in the following equation:

(2) $$\begin{array}{c} D=\frac{K\lambda }{B\;\cos\;\theta }, \end{array}$$

where *D*: the average size of the crystal, *K*: Scherrer's constant, *λ*: wavelength, *B*: the width equivalent to half maximum, and *θ*: position of the peak.

### 2.6. Statistical Examination

The data were examined for normality by Kolmogorov–Smirnov test with SPSS-PC version 21 (SPSS, Armonk, New York, NY, USA). Analysis of variance (ANOVA) was used to determine for the significantly different Vickers microhardness at various phases of evaluation involving baseline hardness (*H*_B_), after acid-activated demineralization (*H*_D_), after the application of remineralizing agent (*H*_A_), with different hardness between demineralization and remineralization (*H*_AD-diff_), and the percentage of hardness recovery (%HR) for demineralized enamel. The Tukey post hoc multiple comparisons were determined for a significant difference in the midst of groups at a 95% level of confidence.

## 3. Results

**Figure 2:**
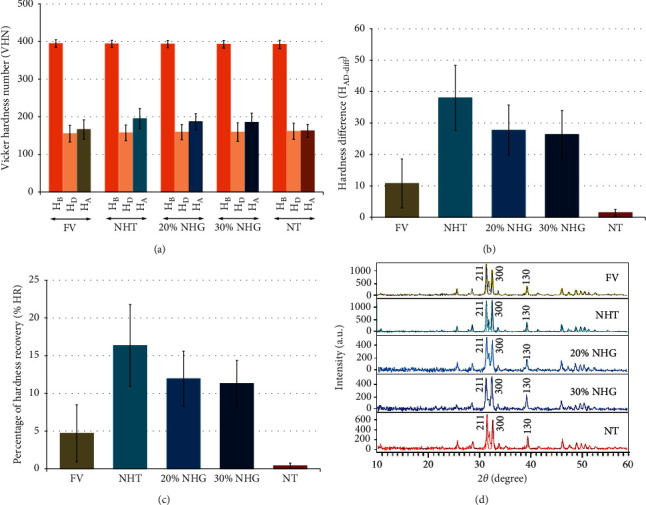
(a) Mean and standard deviation of hardness at baseline (*H*_B_), hardness upon acid-activated demineralized process (*H*_D_), and hardness upon administration of the remineralizing agent (*H*_A_); (b) difference of hardness upon acid-activated demineralization and after application of remineralizing agents (*H*_AD-diff_ = *H*_A_ − *H*_D_); (c) percentage of hardness recovery (%HR); and (d) microscopic crystal structure of each group treated with fluoride varnish (FV), nano-hydroxyapatite toothpaste (NHT), 20% nano-hydroxyapatite gel (20% NHG), and 30% nano-hydroxyapatite gel (30% NHG) compared to no treated group (NT).

The surface hardness at different stages is indicated in Figure 2(a) and Table 2 for each group. No significant difference for *H*_B_ for all evaluated groups (*p* > 0.05) was shown (Tables 3 and 4). The average *H*_D_ was reduced in comparison to *H*_B_ for every group as indicated in Figure 2(a). Nevertheless, no difference was indicated significantly in *H*_D_ between the groups (*p* > 0.05; Tables 3 and 4). Upon the application of the remineralizing materials, the average *H*_A_ was increased significantly in comparison to *H*_D_ for each group, besides the NT. The mean *H*_A_ was a significant difference among groups, except for FV-NT, FV-30% NHG, NHT-20% NHG, NHT-30% NHG, and 20% NHG-30% NHG as indicated in Table 4. Differences in H_AD-diff_ and %HR were significant among the evaluated groups (*p* < 0.05), except between 20% NHG and 30% NHG, as shown in Tables 3 and 4. The %HR was disclosed with the highest mean for the NHT group, followed by 20% NHG, 30% NHG, FV, and NT.

An analysis for XRD revealed crystal structures at the diffraction angle (2*θ*) of 31.7°, 32.9°, and 39.8°, which corresponded with the (211), (300), and (310) planes sequentially for all tested groups as indicated in Figure 2(d). The FV group demonstrated a distinct sharp peak, and the same intensity of peak was detected with the NHT group. The results indicated that both FV and NHT were capable of generating a greater degree of crystalline content in comparison to the other groups. Both groups of 20% NHG and 30% NHG exhibited a wider and lower peak intensity compared with the NHT group, which indicated a combination of an amorphous phase and poorly crystallized apatite. The NT group also detected a broad with short peaks that indicated the establishment of artificial caries lesions, as indicated in Figure 2(d). The crystal size for every group was in the visual range of 34.97 to 41.76 nm. The biggest crystal size was indicated with the NHT group (41.76 nm), accompanied by 20% NHG (39.67 nm), FV (38.62 nm), 30% NHG (36.91 nm), and NT group (34.97 nm) as presented in Table 2.

**Figure 3:**
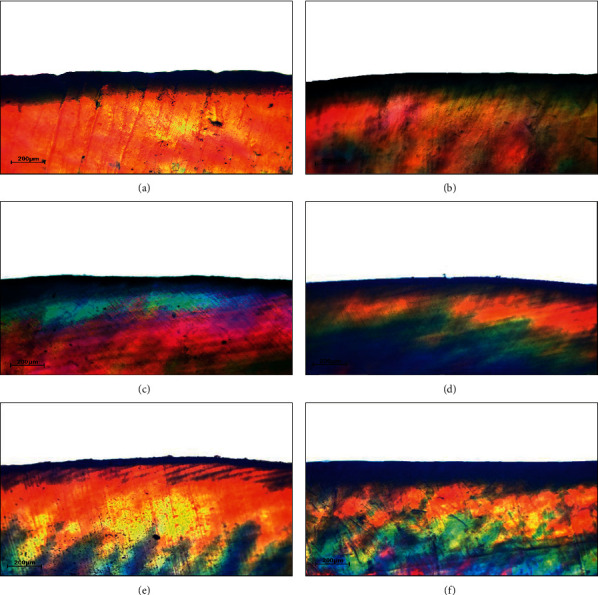
Polarized-light photomicrograph (PLM) of enamel (×10) after demineralization (a), followed by remineralization with fluoride varnish (b) nano-hydroxyapatite toothpaste (c), 20% nano-hydroxyapatite gel (d), 30% nano-hydroxyapatite gel (e) compared to no treatment group (f).

The existence of caries defect and the advancement of the remineralized procedure of everyone group was supported by the PLM, shown in Figure 3, compared to the PLM of the unimpaired enamel not having caries lesion as evidenced in Figure 1(c). The dark zone and advanced depth of lesion were detected in the PLM of the sample with artificial caries (Figure 3(a)). Upon the administration of different remineralizing agents, there was an observed reduction in the depth of lesion for all the treatment groups as shown in Figures 3(b)–3(f). The PLM for both groups of 20% NHG (Figure 3(c)) and 30% NHG (Figure 3(d)), indicated a decrease in the depth of the carious lesion in a similar form as the groups of NHT (Figure 3(e)) and FV (Figure 3(f)).

**Figure 4:**
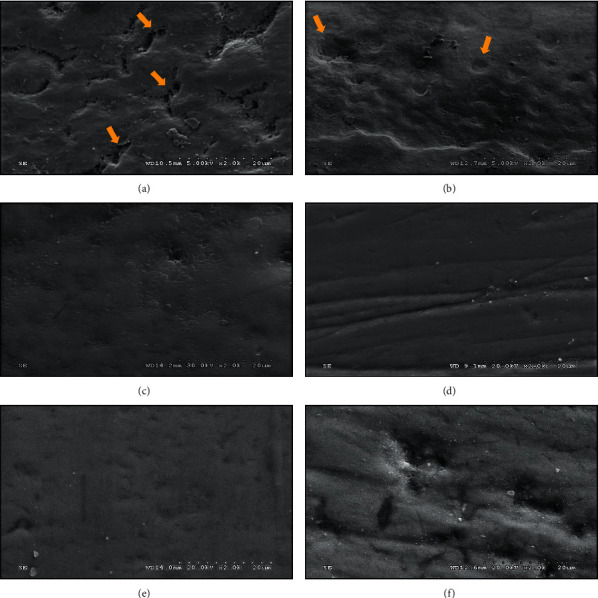
The scanning electron photomicrograph (SEM, x2K) of the demineralized surface of enamel, showing outlines of enamel prism/rod with the remnant of interprismatic enamel (yellow arrow) (a), followed by remineralization with fluoride varnish, showing incomplete filling of the porosities (yellow arrow) (b), nano-hydroxyapatite toothpaste (c), 20% nano-hydroxyapatite gel (d), 30% nano-hydroxyapatite gel (e) compared to no treatment group (f).

The SEM micrograph (×2K) of each tested group is shown in Figure 4(a)–4(f), compared to the SEM micrograph of unimpaired enamel as displayed in Figure 1(d). The induced artificial caries specimen demonstrated a nonuniform pattern of splits and extinction surface, showing excessive porosities as evidenced in Figure 4(a), in comparison with the SEM photomicrograph of even and unimpaired enamel sample as detected in Figure 1(d). Upon application of FV, the SEM photomicrograph indicated a fine film of the mineral deposition process, coupled with an insufficient replenishing of the porosities and voids originated from the formerly generated caries lesion as evidenced in Figure 4(b), that indicated the incompletely remineralized capability of FV on the entire induced carious lesion on the surface of the enamel. The SEM photomicrograph of the NHT specimen indicated a considerably smoother surface than the SEM photomicrograph observed in the group FV as evidenced in Figure 4(c). The SEM photomicrographs for the 20% NHG (Figure 1(d)) and 30% NHG (Figure 1(a)) group displayed a glossy and homogenous surface of enamel identical to the NHT group. The SEM micrographs revealed that a forming of a new layer of apatite and remineralized process occurred after the generated caries lesion of enamel was applied by either NHT or 20% NHG or 30% NHG. On the contrary, profound porosities and intense irregularity pattern of extinction on the SEM micrograph of artificial caries lesion on the surface of enamel for the group of NT were detected as evidenced in Figure 4(f).

## 4. Discussion

The concept of remineralization on initial caries lesions is a preventive means in conservative dentistry for the next decade. This in vitro study attempted to evaluate the capabilities of FV, NHT, 20% NHG, and 30% NHG in the remineralization of enamel caries. The result of the study suggested that either FV or NHT or 20% NHG or 30% NHG were significantly capable of remineralization proficiency to recover the demineralized enamel, compared with the nontreated demineralized surface. Therefore, the null hypothesis was rejected. This means that the remineralization potential was a significant difference among the remineralization products used in this study. The NHT indicated significantly better capacity to remineralize the demineralized enamel surface than either NHG or FV as evidenced from SEM and indicated more crystalline particles deposition in the remineralization area as well as higher increasing XRD peak intensity of crystalline particles in NHT group than other groups.

This study used FV as a positive control. The varnish was developed to prolong the contacting time between the fluoride and enamel surface and slowly release calcium fluoride (CaF_2_), forming a reservoir to be ready for deposition in the crystal structure of the carious lesion [7]. The firmly bound fluoride on the enamel structure known as fluorapatite is the most beneficial in anticaries efficacy. This study indicated that FV was capable of enhancing hardness to carious enamel as the experiment was designed focusing on the potential remineralization of the fluoride only from the FV agent by immersing the specimen in the deionized water without other sources of fluoride, leading to the result that was supported by other studies [8, 9]. However, the remineralization capability of FV in the present study was lesser than NHT and NHG for both concentrations. This is possibly related to the limited contact time of FV that might exhibit only a partial chemical reaction not enough to fill up in the structure of the artificially induced carious enamel. In addition, the fluoride complex found in the enamel can be easily decreased after application [4]. Therefore, the surface microhardness presented in this study was lesser compared to other mineralization products.

The remineralization potential of nano-HA products showed a superior capability than FV and the control group due to its smaller-sized particles. The nano-size of particles and greater functioning of the nano-HA possibly enable either calcium or phosphate ions available in the nano-HA to be proficient in penetrating the enamel surface and continue filling the porosities of artificial carious lesions better than fluoride [22]. This study indicated that the nano-HA for both NHT and NHG for either 20% and 30% in concentration was comparable to increasing the surface microhardness for carious enamel. Yet the capability of remineralization of NHT was greater than NHG for both concentrations as the recovery of hardness from the carious lesion was higher for NHT compared to NHG, which is possibly related to the better stability of the toothpaste product in the remineralization process than NHG.

This study showed that 20% NHG was equally capable of remineralization to 30% NHG. This might indicate that increase in the concentration of NHG might not be the prime factor indicating the remineralization potential for carious lesions. This possibly related to the method of product preparation for nano-HA that affected its remineralization potential. Previous studies suggested that 10% nano-HA was the optimal concentration for effective remineralization [23]. Most studies have used nano-HA in slurry preparation [4, 22], while this study prepared the product as an experimental gel using SCMC as a vehicle for nano-HA. The use of SCMC was intended to increase the viscosity of the gel to provide a slower rate of diffusion of minerals into the carious surface of enamel, which was supported by other studies [31]. We assumed that a higher concentration of nano-HA together with SCMC might compensate for the slow rate of diffusion of nano-HA. Thus, it might result in better increasing surface microhardness. Nonetheless, the results of our study showed that an increasing concentration of NHG was not capable of enhancing hardness recovery over the NHT. This is possibly related to the plateauing effect that could be observed in the high concentration of nano-HA as a result of the aggregation of particles on the enamel surface.

The other important constituent that may affect the potential in remineralization of the nano-HA is the pH value of the prepared solution. It was assumed that pH values higher than 5.5 would arrest caries progression and might promote remineralization [29]. However, in a previous study, an acidity circumstance would intensify the soluble ability of the nano-HA, thus enhancing the precipitation of nano-HA onto the surface of enamel [22]. In this present study, the NHT has lower pH of 5.83 compared to the 20% and 30% NHG with an average pH of 9.86 and 9.57, respectively. This supported the results of the superior remineralization effect on the percentage of hardness recovery for NHT compared to NHG.

All tested groups, excluding the negative control group, showed the depreciation of the depth of caries lesion as evidenced from the PLM photomicrograph. Nonetheless, the completed remineralized process was not accomplished in this study. This was presumed that the hydroxyl radical was displaced by the fluoride ion, the outermost layer of surface developed into intense exothermic condition, whilst the innermost layer gradually generated inferior exothermic, thus conveying to replacing the hydroxyl radical on the surface with fluoride ions comfortably [14]. Hence, the outermost layer of enamel surface turned into extremely mineralized, commonly named “hyper-mineralization,” leading to the incapability of minerals to penetrate the deeper layers. This hypermineralization effect was observed from the photomicrograph of PLM upon the group FV in this study. This effect was observed in the groups of NHT, 20% NHG, and 30% NHG as evidence from the PLM photomicrograph, which was supported by other studies [23]. In addition, the high initial rate of deposition of minerals on the surface layer might slow down and inhibit the diffusion of ions into the deeper regions of the lesion thus forming a hypermineralized layer. As such, it was suggested that combining nano-HA with other elements or natural products such as Galla Chinensis may overcome the limitation of diffusion and create a synergism effect to improve the remineralization potential of nano-HA [32].

The SEM micrographs indicated that the NHT and NHG were capable of forming a homogenous surface of the apatite layer that was confirmed by previous studies [20]. A study showed that during 10 minutes of treatment in a slurry preparation of nano-HA, the enamel was surrounded with a homogenous layer by chemically attaching synthetic HA to the enamel surface that led to the establishment of a layer of new apatite [13]. An intense connection of the nano-HA to the enamel surface can be achieved owning to the calcium and phosphate crystallinity where they illustrated an important part in the mineralization of the enamel [22]. Moreover, a new nano-HA layer was observed to be insensitive to demineralization [4, 26]. On the other hand, fluoride varnish showed an incomplete filling of the porosities due to the limited fluoride available for calcium and phosphate binding rather than affecting the calcium phosphate levels on the enamel [13, 26]. This structural binding could only inhibit the solubility of the enamel and not be able to reconstruct mineral loss from demineralization, as supported by the result of this study.

The detected peaks of all tested groups upon XRD evaluation revealed an identical crystalline structure to naturally appeared enamel. For the FV group, the hydroxyapatite and fluorapatite were difficult to distinguish as the peak positions were minor differences [24, 25]. The intense peaks detected in the FV group suggested extreme crystallinity that is possibly owing to the alteration of crystalline hydroxyapatite to crystalline fluorapatite [26]. On the other hand, the short and broadened peaks of 20% and 30% nano-HA gel could be observed in our study that indicated less crystallinity compared to the narrow and sharp peaks of nano-HA toothpaste. The differences in the peak intensities of the nano-HA gel and nano-HA toothpaste also signified a smaller crystal size of the gel configuration, which was endorsed by Scherrer's equation. The stretching peak detected from the NT group was probably associated with the partially dissolved crystals, rendering the amplification of the intercrystalline spaces, thus diminishing their crystal sizes [37].

This study indicated that nano-HA gel could be considered as the treatment alternative for remineralization therapeutics in the high-risk group of patients who are unable to control their oral hygiene practices by conventional methods of tooth brushing and/or limited access to fluoride varnish application provided by oral health care workers. Therefore, the nano-HA gel can then be developed as a novel product that may be more cost-effective and be a potential treatment option to treat dental caries in this special group of patients. Within the limitation of the study design on ideal clinical simulation, the authors are confident that the results of the study would have solely an impact on the use of hydroxyapatite gel on early remineralization of initial carious lesion on the surface of dental enamel. Nevertheless, clinical investigation needs to be performed in the future to validate the results of this preliminary study on remineralization potential of nano-hydroxyapatite gel, as well as further evaluation of the nano-HA gel such as Ca/P ratio analysis. The properties and characteristics of the gel must maximize the benefits and strength of using nano-HA in remineralization.

## 5. Conclusion

The present study indicated that nano-HA gel either in 20% or 30% concentrations was capable of remineralization for the artificial caries lesion, and both will be more challenging in further clinical studies. The nano-HA gel application was effective in increasing the microhardness, decreasing the depth of the lesion, and developing a unique apatite layer of enamel comparable to the commercially available nano-HA toothpaste. Nano-HA both in toothpaste and gel form was capable of remineralization better than fluoride varnish.

## 6. Clinical Significance

Remineralization is a state-of-the-art treatment approach for caries lesions by detecting at the early phase of the disease and rendering a noninvasive therapeutic strategy, which is essential for the dental health professional in shifting to a modern paradigm of treatment. This present study manifested that a topical application of nano-HA gel indicates an attractive route to deliver the material in the oral cavity and can be more effective and less toxic than conventional formulations and provide its effectiveness directly at the site of action, especially for the noncooperative young child and the medically compromised patient who faced with trouble in using toothbrushes and toothpaste for hygiene control.

## Figures and Tables

**Table 1:** Materials, compositions, company, and their average pH values of remineralizing agents evaluated in this present study.

Material	Company	Composition	pH (mean ± sd)
Fluoride varnish (FV)	Duraphat®, Colgate Palmolive Co., Guildford, UK	2.26% Sodium fluoride, ethanol, colophonium, matrix, shellac, wax, saccharine, flavor	7.0

Nano-HA toothpaste (NHT)	Apagard®, Sangi Co., Tokyo, Japan	Water, dental-grade dibasic calcium phosphate, concentrated glycerin, medical hydroxyapatite, Macrogol 400, *β*-glycyrrhetinic acid, cetylpyridinium chloride, silicic silicate, sodium lauryl sulfate, sodium carboxymethyl cellulose (SCMC), flavor, hydrolyzed conchiolin solution, saccharin sodium, trimagnesium phosphate, alkyldiaminoethylglycine hydrochloride solution	5.83 ± 0.03

20% Nano-HA gel (20% NHG)	Pediatric Dentistry and Biomaterials Research, KKU	Nano-HA powder, deionized water, sodium carboxymethyl cellulose (SCMC)	9.86 ± 0.06

30% Nano-HA gel (30% NHG)	Pediatric Dentistry and Biomaterials Research, KKU	Nano-HA powder, deionized water, sodium carboxymethyl cellulose (SCMC)	9.57 ± 0.02

**Table 2:** Mean ± standard deviation (sd), 95% confidence interval (CI) of hardness at baseline (*H*_B_), hardness upon acid-activated demineralization (*H*_D_), hardness after application of remineralizing agent (*H*_A_), hardness difference determined between after acid-activated demineralization and after application of remineralizing agents (*H*_AD-diff_ = *H*_A_ − *H*_D_), percentage of hardness recovery (%HR) of each group after application of remineralizing agents (*H*_A_) in comparison to after acid-activated demineralization (*H*_D_), and average crystal size for each treatment group.

Treatment group	*n*	*H*_B_	*H*_D_	*H*_A_	*H*_diff_ = *H*_A_ − *H*_D_	%HR	Crystal size (nm)
		Mean ± sd 95% CI (LL–UL)	Mean ± sd 95% CI (LL–UL)	Mean ± sd 95% CI (LL–UL)	Mean ± sd 95% CI (LL–UL)	Mean ± sd 95% CI (LL–UL)	
Fluoride varnish (FV)	20	394.72 ± 10.00^a^ (390.04–399.40)	155.29 ± 22.16^a^ (144.93–165.66)	166.06 ± 25.93^a^ (153.93–178.19)	10.77 ± 7.80^*∗*^ (7.12–14.41)	4.71 ± 3.76 (2.94–6.48)	38.62

Nano-HA toothpaste (NHT)	20	393.97 ± 9.64^a^ (389.45–398.48)	157.21 ± 21.14^a^ (147.31–167.10)	195.21 ± 26.72^b^ (182.71–207.71)	38.00 ± 10.33^*∗*^ (33.17–42.84)	16.34 ± 5.43 (13.80–18.89)	41.76

20% Nano-HA gel (20% NHG)	20	393.57 ± 9.76^a^ (389.00–398.13)	159.37 ± 20.02^a^ (150.00–168.74)	187.09 ± 21.41^c^ (177.07–197.11)	27.72 ± 7.99^*∗*^ (23.98–31.46)	11.93 ± 3.65 (10.22–13.64)	39.67

30% Nano-HA gel (30% NHG)	20	392.96 ± 10.38^a^ (388.10–397.82)	158.99 ± 24.80^a^ (147.34–170.64)	185.32 ± 24.45^d^ (173.87–196.76)	26.33 ± 7.64^*∗*^ (22.75–29.90)	11.31 ± 3.04 (9.88–12.73)	36.91

No treatment (NT)	20	392.50 ± 11.09^a^ (387.31–397.69)	161.43 ± 21.56^a^ (151.34–171.53)	162.92 ± 16.95^a^ (154.99–170.86)	1.50 ± 10.22^$^ (−3.29–6.28)	0.40 ± 4.34 (−1.63–2.44)	34.97

Similar letters represent no statistical difference within the same period in different treatments by ANOVA (*p* > 0.05). ^*∗*^*p* < 0.05; ^$^*p* > 0.05.

**Table 3:** Analysis of variance (ANOVA) of hardness at baseline (*H*_B_), hardness upon demineralized process (*H*_D_), hardness upon an applied remineralizing agent (*H*_A_), hardness difference determined between after demineralization and after remineralization (*H*_AD-diff_ = *H*_A_ − *H*_D_), and percentage of hardness recovery (%HR) after applied remineralizing agents (*H*_A_) compared to demineralization (*H*_D_).

*A. ANOVA of hardness at baseline (H*_*B*_)					
Source	SS	df	MS	*F*	*p*
Between groups	59.624	4	14.906	0.144	0.965
Within group	9,862.904	95	103.820		
Total	9,922.527	99			

*B. ANOVA upon acid-activated demineralized process (H*_*D*_)					
Source	SS	df	MS	*F*	*p*
Between groups	429.841	4	107.460	0.222	0.926
Within group	46,035.366	95	484.583		
Total	46,465.207	99			

*C. ANOVA upon administration of the remineralizing agent (H*_*A*_)					
Source	SS	df	MS	*F*	*p*
Between groups	15,869.618	4	3,967.405	7.267	0.001
Within group	51,862.680	95	545.923		
Total	67,732.298	99			

*D. ANOVA for the difference of hardness between demineralization and remineralization (H*_*AD-diff*_)					
Source	SS	df	MS	*F*	*p*
Between groups	16,952.678	4	4,238.169	53.763	0.001
Within group	7,488.908	95	78.831		
Total	67,966.394	100			

*E. ANOVA of the percent of the recovery of hardness (%HR)*					
Source	SS	df	MS	*F*	*p*
Between groups	3,203.24	4	800.81	47.061	0.001
Within group	1,616.57	95	17.017		
Total	4,819.813	99			

SS: sum of squares, df: degree of freedom, MS: mean square, *F*: *F*-ratio, and *p*: *p* value

**Table 4:** Tukey's post hoc multiple comparisons of hardness at baseline (*H*_B_), hardness upon demineralized process (*H*_D_), hardness upon an applied remineralizing agent (*H*_A_), hardness difference determined between after demineralization and after remineralization (*H*_diff_ = *H*_A_ − *H*_D_), and percentage of surface microhardness recovery (%HR) after applied remineralizing agents (*H*_A_) compared to demineralization (*H*_D_) for each treatment group.

*A. Tukey HSD multiple comparisons of hardness at baseline (H*_*B*_)					
Group	FV	NHT	20% NHG	30% NHG	NT
FV	1	0.999	0.996	0.982	0.959
NHT		1	1.000	0.998	0.991
20% NHG			1	1.000	0.997
30% NHG				1	1.000
NT					1

*B. Tukey HSD multiple comparisons after acid-activated demineralization (H*_*D*_)					
Group	FV	NHT	20% NHG	30% NHG	NT
FV	1	0.999	0.977	0.984	0.903
NHT		1	0.998	0.999	0.974
20% NHG			1	1.000	0.998
30% NHG				1	0.997
NT					1

*C. Tukey HSD multiple comparisons after application of remineralizing agent (H*_*A*_)					
Group	FV	NHT	20% NHG	30% NHG	NT
FV	1	0.001	0.042	0.077	0.993
NHT		1	0.806	0.668	0.001
20% NHG			1	0.999	0.013
30% NHG				1	0.025
NT					1

*D. Tukey HSD multiple comparisons of hardness difference (H*_*AD-diff*_)					
Group	FV	NHT	20% NHG	30% NHG	NT
FV	1	0.001	0.001	0.001	0.012
NHT		1	0.004	0.001	0.001
20% NHG			1	0.998	0.001
30% NHG				1	0.001
NT					1

*E. Tukey HSD multiple comparisons of percent for hardness recovery (%HR)*					
Group	FV	NHT	20% NHG	30% NHG	NT
FV	1	0.001	0.001	0.001	0.012
NHT		1	0.009	0.002	0.001
20% NHG			1	0.989	0.001
30% NHG				1	0.001
NT					1

## Data Availability

The data supporting the findings for this study are included in the article.
